# Low validity of the Sensewear Pro3 activity monitor compared to indirect calorimetry during simulated free living in patients with osteoarthritis of the hip

**DOI:** 10.1186/1471-2474-15-43

**Published:** 2014-02-19

**Authors:** Andreas Hermann, Mathias Ried-Larsen, Andreas Kryger Jensen, René Holst, Lars Bo Andersen, Søren Overgaard, Anders Holsgaard-Larsen

**Affiliations:** 1 Orthopedic Research Unit, Department of Orthopaedic Surgery and Traumatology, Odense University Hospital, Odense, Denmark; 2 Institute of Clinical Research, University of Southern Denmark, Odense, Denmark; 3 Department of Orthopaedic Surgery, Herlev University Hospital, Copenhagen, Denmark; 4 Institute of Sport Sciences and Clinical Biomechanics, University of Southern Denmark, Odense, Denmark; 5 Department of Biostatistics, Institute of Public Health, University of Southern Denmark, Odense, Denmark; 6 Institute of Regional Health Research, University of Southern Denmark, Odense, Denmark; 7 Department of Orthopaedic Surgery, Herlev University Hospital, Herlev Ringvej 75, 2750 Herlev, Denmark

## Abstract

**Background:**

To validate physical activity estimates by the Sensewear Pro3 activity monitor compared with indirect calorimetry during simulated free living in patients diagnosed with osteoarthritis of the hip pre or post total hip arthroplasty.

**Methods:**

Twenty patients diagnosed with hip osteoarthritis (10 pre- and 10 post total hip arthroplasty; 40% female; age: 63.3 ± 9.0; BMI: 23.7 ± 3.7). All patients completed a 2 hour protocol of simulated free living with 8 different typical physical activity types. Energy consumption (kcal/min) was estimated by the Sense Wear pro3 Armband activity monitor and validated against indirect calorimetry (criterion method) by means of a portable unit (Cosmed K4b^2^). Bias and variance was analyzed using functional ANOVA.

**Results:**

Mean bias during all activities was 1.5 Kcal/min 95%CI [1.3; 1.8] corresponding to 72% (overestimation). Normal gait speed showed an overestimation of 2.8 Kcal/min, 95%CI [2.3; 3.3] (93%) while an underestimation of -1.1 Kcal/min, 95%CI [-1.8; -0.3] (-25%) was recorded during stair climb. Activities dominated by upper body movements showed large overestimation with 4.37 Kcal/min, 95%CI [3.8; 5.1] (170%) being recorded during gardening. Both bias and variance appeared to be dependent on activity type.

**Conclusion:**

The activity monitor generally overestimated the energy consumption during common activities of low to medium intensity in the patient group. The size and direction of the bias was highly dependent on the activity type which indicates the activity monitor is of limited value in patients with hip osteoarthritis and that the results do not express the real energy expenditure.

## Background

Patients with osteoarthritis (OA) of the hip have excess all cause mortality including increased mortality related to cardiovascular disease which has been associated with reduced patient reported physical activity (PA) [1]. Studies indicate that a majority of patients with lower extremity OA may not meet general recommendations regarding PA [2]. However, as the same is evident for the elderly population in general [3] it is still uncertain to which extend the functional impairment and pain present in symptomatic hip OA affects the actual PA compared with the general population. Studies of the objectively measured PA in patients with hip OA and total hip arthroplasty (THA) are limited especially regarding comparison with healthy controls [4-6] and their results are restricted by the general lack of validation studies of the used data collecting tools applied in patients with degenerative joint disease. Thus, establishing knowledge of the validity of objective measured PA in hip osteoarthritis patients is of importance for future research.

PA is defined as any bodily movements produced by skeletal muscles that require energy expenditure [7]. Energy expenditure during PA is commonly investigated by indirect calorimetry which requires either isolation of individuals in closed spaces or portable apparatus for gas analysis of air exchange [8,9]. These methods are often referred to as criterion methods [10] but their application is limited to small laboratory settings [8,9]. In free living small body-worn multisensory activity monitors based on accelerometry can be used as a feasible surrogate measure of energy expenditure during PA [11-13]. In clinical studies such activity monitors are applied due to their objectivity compared to self-reported physical activity questionnaires [5,14] and they may become a tool in future etiological and prognostic studies in patients with lower extremity osteoarthritis [15]. However, in hip OA patients altered movement patterns may occur [16,17] and functional impairment and pain may affect the speed of exercises both potentially affecting estimations of energy expenditure based on accelerometry.

In the current study the Sensewear pro3 (SWA) activity monitor armband was validated. The SWA is a small multisensory activity monitor that combines accelerometry with various physiological data (see Method; Equipment). The monitor requires minimal instruction in use which suits the application in free living studies. The outcome in terms of energy expenditure is readily comparable with recommendations for PA (e.g. The American College of Sports Medicine [18]). Recently, the SWA has been used in a various clinical studies of actual PA in different patient groups [14,19-23] and in OA patients the monitor has been applied in a comparable study of PA between patients with hip and knee OA and healthy controls [5]. However, like other activity monitors the validity in this patient group is unknown. Varying degrees of bias has been observed when validated in healthy older adults [11,24], obese adults [25] and in various patient groups including patients with rheumatoid arthritis [13,26-28]. In healthy adults the SWA been reported reliable [25,29] and valid for estimation of cumulated daily energy expenditure [12,30]. However, limitations regarding validity has been reported during various activity types [31-34].

The purpose of this study was to investigate the validity of the SWA activity monitor in patients with hip osteoarthritis during a simulated free living protocol according to the following 3 proprieties: i) Bias between activity monitor estimates and indirect calorimetry (criterion method), ii) correlation between methods and iii) difference in variance [35].

## Method

### Participants

A convenience sample of 20 patients (10 of preoperative stage, 10 of postoperative stage) diagnosed with hip OA (Gender: 40% female, Age: 63.3 ± 9.2 years, BMI: 23.7 ± 3.8) treated with THA or scheduled for THA at the Department Orthopaedic Surgery and Traumatology, Odense University Hospital (12 males, 8 females), were included.

### Inclusion/exclusion

*Inclusion criteria* for the preoperative group: Diagnosed primary OA of the hip and scheduled for surgery (THA).

*Inclusion criteria* for the postoperative group: Diagnosed primary OA of the hip, treated with THA within 6 to 12 months of inclusion.

*Exclusion criteria* (both groups): Patients with a known history of symptomatic lung or heart disease or known symptoms of claustrophobia or unease using a mask and patients not understanding Danish language were excluded. Patients dependent on walking aid (and therefore unable to comply with the free living protocol) were excluded as well. Finally, for the post surgery group, patients with a scheduled reoperation of the hip or previous dislocation were excluded.

Twenty five were asked, 3 declined to participate and 1 was excluded due to known symptomatic lung disease and 1 due to known symptoms of claustrophobia. All 20 participants were able to complete the free living scenario.

All participants gave informed written consent and the conditions and methods of the study protocol was approved by the Ethical Committee, Region of Copenhagen, Denmark (Identifier; H-2-2010-47) and performed in accordance with the Helsinki Declaration of 1975, as revised in 2000.

### Equipment

The activity monitor:

A small multisensory activity monitor (Sensewear Pro3 armband (SWA)) was positioned over the triceps brachii muscle of the right arm at the midpoint between the acromion and olecranon processes (size; 85.3 mm × 53.4 mm × 19.5 mm). The activity monitor collects physiological data from following sensors; a 2 axial accelerometer, a heat flux sensor, a skin temperature sensor, a near body ambient temperature sensor, and a galvanic skin response sensor. The activity monitor uses an onboard algorithm (InnerView TM Professional software version 5.1.0) fitted with anthropometric data from the participant (gender, age, height, and weight). The output is energy expenditure (kcal/min) calculated by an internal inaccessible algorithm.

Criterion Method:

Indirect calorimetry: For validation of the SWA armband a portable metabolic monitor (Cosmed model K4b^2^) was worn during the protocol. The K4b^2^ weighs 1.5 kg including a battery and is mounted on the chest with a simple harness. The K4b^2^ has been shown valid in comparison to Douglas bag method [36]. Prior to the study the apparatus had been serviced by the manufacture and validated against Douglas Bag by the authors (Data not shown). Before each test, the monitor was calibrated in accordance with the manufactures instructions. Energy expenditure (kcal/min) was calculated from the breath-by-breath oxygen use and carbon monoxide production.

### Study protocol

A two hour protocol of 8 activities of daily living was designed. Activities imitate common activities of daily living expected for the patient/age group. Activities were: I) rest; 53 minutes (which includes all periods of rest in sitting and supine position), II) a simple warm-up program with steps and multi-planar movements; 9 minutes, III) sitting and walking between chairs; 4 minutes, IV) ascending and descending stairs; 4 minutes (4 steps, step height 15 cm), V) walking; normal; 15 minutes (self-paced) and brisk walking; 10 minute, VI) jogging; 5 minutes (or brisk walking), VII) outdoor gardening; 10 minutes (raking), and VIII) indoor cleaning; 10 minutes (sweeping floor).

All activities were supervised and performed in a consecutive order following the protocol without time breaks or discontinuity of measurements. Participants were instructed to perform the activities within the intensities of their daily living. If an activity was impossible to perform due to pain or impairment of hip movements a lower intensity level was selected and the alteration was registered.

Subjects were fasting and refrained from smoking and drinking coffee 1 hour prior to testing to diminish possible influence on the basic energy expenditure. Before each assessment, the activity monitor was initialized and fitted to the patient according to the manufacturer’s instruction. The data was downloaded in 1 minute epochs by software provided by the manufacturer (InnerView Professional Research Software Version 5.1.0).

The K4b2 was calibrated and mounted on the participant. For acclimatization the subjects rested seated 10 minutes prior to the protocol. To identify the time periods of the individual activities during the later data analysis both units (the SWA and the K4b2) and the time scheme of the protocol were synchronized by an electronic clock. The validation procedure including the initial calibration of units was performed by the principal author.

### Data analysis

Bias was defined as the difference between the activity monitor and indirect calorimetry outcomes (kcal/min). Activity specific bias was analyzed for each activity separately (the 15 time intervals coded #1-#15). To diminish possible carry over effects between intervals due to VO_2_ latency, the first minute of each interval (#1-#15) was excluded from the later mean bias analysis of each activity and intervals of 2 minutes and less (interval #3 and #5) were regarded non-conclusive results. Mean bias of all 15 intervals (#1-#15) are presented.

### Statistical analysis

Statistical analysis was carried out using functional data analysis [37]. This approach treats an entire curve of observations as a single datum rather than a collection of separate observations. In the present context each time dependent trajectory of the activity monitor and indirect calorimetry represents an observation. The techniques allow for a flexible characterization of the dynamics with minimal assumptions. In contrast, traditional methods such as linear mixed models that are based on the individual time points impose a parameterization on the functional form of the mean.

Specifically, we are interested in estimating the first two functional moments of the data. The functional mean leads to the definition of a time dependent bias function that varies freely over durations of the activities.

From the second order moments the functional variance processes [38] and the correlation coefficient were estimated [39] where the former characterize the internal stability of the activity monitor and indirect calorimetry.

The first step was to project the observed data into function space. We used a cubic b-spline basis with a knot placed at every minute and a data adaptive roughness penalty on the second derivative. The penalty parameter was estimated using the generalized cross-validation criterion [37].

A two-way functional ANOVA model showed no significant effect of surgical status, thus this factor was removed and the following results are based on pooled data.

The bias function was estimated as the functional mean of the pair-wise differences between the activity monitor and the indirect calorimetry curves with corresponding 95% confidence bands estimated by the method described by Cuevas et al. (2006) using the L2 norm as proximity measure [40].

The mean and relative biases of each interval (#1-#15) was calculated by a numeric quadrature rule over the corresponding intervals and the confidence intervals were based on a pair-wise re-sampling procedure.

Statistical analysis was carried out using R version 2.15.2 (2012-10-26) “Trick or Treat” Copyright (C) 2012 The R Foundation for Statistical Computing ISBN 3-900051-07-0.

## Results

Descriptive characteristics of subjects are shown in Table 1.

**Table 1:** Subject characteristics

	**Total (n = 20)**	**Female (n = 8)**	**Male (n = 12)**
Age (years)	63.3 ± 9.2	67.1 ± 8.6	60.7 ± 9.0
Weight (kg)	82.8 ± 15.0	73.4 ± 11.2	89.0 ± 14.3
Height (m)	174.2 ± 7.7	167.8 ± 5.2	178.5 ± 5.9
BMI (kg/m^2^)	23.7 ± 3.8	21.9 ± 3.3	24.9 ± 3.8

Data are $\bar{x}\pm \mathrm{SD}$.

All participants completed the protocol and all activities were performed according to the protocol except during activity #13 in which all participants declined to perform jogging due to self esteemed lack of physical capability. Brisk walking was performed instead.

**Figure 1:**
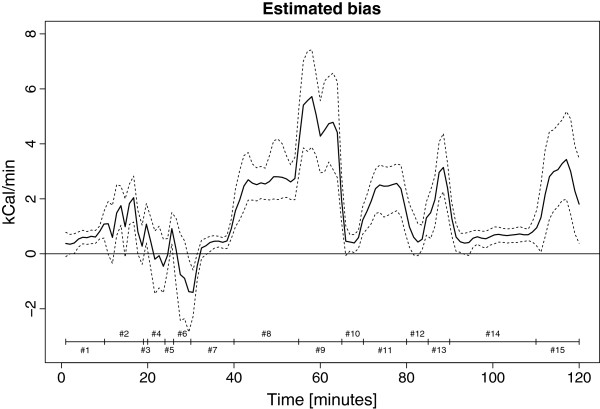
**Bias between Sensewear Pro3 (SWA) estimates and indirect calorimetry (gold standard).** Bias expressed as the mean difference with 95% confidence intervals. The horizontal line represents no difference between the methods. A positive value represents an overestimation of SWA. Coding #1-#15 represents intervals of steady state activity (see Table 2).

Figure 1 illustrates the mean bias (difference) between the SWA and indirect calorimetry as a continuous time function with 95% confidence intervals. The bias is mainly significant positive (overestimation) with a fluctuant pattern that appears to follow transitions in activity mode.

The total energy expenditure was overestimated by 72% by the SWA during giving a significant average overestimation of 1.5 Kcal/min, 95%CI (1.3,1.8) during all activities (Table 2).

**Table 2:** Activity types of the protocol with coding for intervals

**Activity type**	**Length (min)**	**Interval**	**EE SWA (kcal/min)**	**EE IC (kcal/min)**	**Bias (kcal/min)**	**Bias (%)**
**Total**	**120**		**3.7 [3.4; 4.0]**	**2.2 [1.8; 2.6]**	**1.54 [1.3; 1.8]**	**71.8 [51.7; 92.8]**
**Resting in chair**	10	#1	1.5 [1.4; 1.6]	0.9 [0.7; 1.1]	0.6 [0.5; 0.8]	77.8 [45.2; 117.5]
**Work out (steps and multi planar movements)**	9	#2	4.2 [3.8; 4.6]	3.0 [2.5; 3.5]	1.2 [0.7; 1.6]	40.3 [21.0; 60.8]
**Resting in chair**^**2**^	1	#3	3.0 [2.6; 3.4]	2.3 [1.9; 2.7]	0.7 [0.1; 1.2]	29.6 [5.4; 57.3]
**Sitting/standing and walking between 2 chairs**	4	#4	3.6 [3.1; 4.1]	3.8 [3.2; 4.4]	-0.2 [-0.8; 0.4]	-4.7 [-19.6; 10.5]
**Resting I chair**^**2**^	2	#5	2.6 [2.0.; 3.1]	2.1 [1.6; 2.5]	0.5 [0.1; 1.0]	27.0 [2.4; 59.3]
**Stair climbing (5 steps up/down)**	4	#6	3.1 [2.7; 3.6]	4.2 [3.6; 4.9]	-1.1 [-1.8; -0.3]	-24.8 [-39.1; -7.6]
**Resting in a supine position**	10	#7	1.5 [1.3; 1.6]	1.0 [0.8; 1.2]	0.5 [0.3; 0.7]	53.1 [25.6; 81.0]
**Walking normal speed (self paced)**	15	#8	5.8 [5.1; 6.5]	3.0 [2.5; 3.5]	2.8 [2.3; 3.3]	93.3 [72.0; 119.1]
**Outdoor gardening (raking leaves)**	10	#9	7.0 [6.1; 7.8]	2.6 [2.2; 3.1]	4.4 [3.8; 5.1]	170.3 [134.0; 211.4]
**Resting in chair**	5	#10	1.8 [1.6; 2.0]	1.0 [0.8; 1.3]	0.8 [0.5; 0.9]	73.9 [42.5; 105.7]
**Brisk walking**	10	#11	5.7 [5.2; 6.2]	3.5 [2.9; 4.1]	2.2 [1.7; 2.6]	62.9 [42.3; 87.2]
**Resting in chair**	5	#12	2.1 [1.8; 2.4]	1.2 [1.0; 1.5]	0.9 [0.6; 1.2]	71.8 [41.3; 107.7]
**Jogging/brisk walking**	5	#13	6.1 [5.2; 7.2]	3.8 [3.1; 4.5]	2.3 [1.8; 2.9]	61.9 [45.3; 81.9]
**Resting in chair**	20	#14	1.4 [1.3; 1.5]	0.8 [0.6; 1.0]	0.7 [0.5; 0.8]	88.0 [47.2; 136.2]
**Sweeping floor**	10	#15	5.0 [4.2; 5.8]	2.3 [1.9; 2.8]	2.7 [1.9; 3.5]	119.4 [75.0; 172.1]

Mean values of energy expenditure (EE) measured by Sensewear Pro3 (SWA) and indirect calorimetry (IC) and absolute and relative bias between units^1^. Positive bias values indicate overestimation of SWA.

^1^Values are $\bar{x}$ with 95% confidence interval.

^2^Regarded as non conclusive due do short time period (see text).

During walking activities (#8, #11, #13) overestimation ranged between 62% and 93%. Significant underestimation (-25%) was observed during ascending/descending stairs (#6) while intervals dominated by upper body movement (#9 and #15) showed large overestimation of 170% and 119% for outdoor gardening and indoor cleaning, respectively (Table 2).

**Figure 2:**
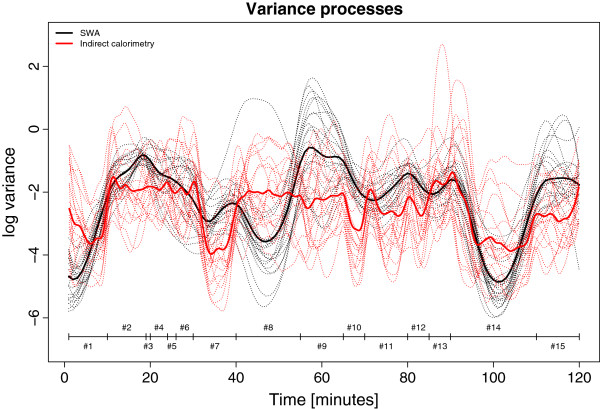
**Functional variance processes of the Sensewear pro3 (SWA) and indirect calorimetry measurements showing the internal stability.** Lower values indicate higher internal stability. Coding #1-#15 represents intervals of steady state activity (see Table 2).

Figure 2 illustrates the variance processes of the two methods and demonstrates the SWA to be less stable during most activities except for periods of resting. The correlation coefficient between methods (all activities) was 0.94.

## Discussion

In the present study the SWA activity monitor was validated during simulated activities of daily living in a group of patients with hip osteoarthritis before or after THA by means of indirect calorimetry. The main findings were significant overestimations of energy expenditure by the activity monitor of up till 170% during common activities of daily living. Bias and variance showed dependency on the type of activity performed.

The SWA has been used for estimation of PA in various patient groups including patients with hip and knee OA [5,14,19-23]; however, to our knowledge no previous studies have investigated the validity of the SWA or other activity monitors in patients with OA of the hip. The majority of validation studies of have been conducted in healthy adults [12,30-34,41] of which two studies have reported the SWA as a valid tool for estimation of cumulated daily energy expenditure in comparison with doubly labeled water [12,30]. This contrasts with the majority of the activity specific protocols (using indirect calorimetry as criterion method) reporting the validity to be dependent of both the intensity and type of activity [31,33,34,41,42]. Direction of bias during walking activities may change according to inclination [34] and overestimation has been reported during exercise of the upper extremities [31]. This is in correspondence with the current findings of underestimation during stair climbing activities and overestimation during horizontal walking and in activities dominated with upper body movements. In healthy elderly numbers of validation studies are few and inconclusive in particular regarding the validity during activities [11,24]. In a study of resting energy expenditure in healthy elderly individuals (age (years); males 67.9 ± 5.1, females 69.2 ± 5.1) Heiermann et al. (2011) found an overestimation (12-14%) compared to indirect calorimetry [24] while Mackey et al. (2011) reported the SWA to be a valid tool for estimation of cumulated daily energy expenditure compared to doubly labeled water. Activity specific protocols in healthy elderly are currently lacking. In our population (age (years); 63.3 ± 9.2) we observed overestimation during rest and in the majority of activities (Table 2). Despite difficulty in comparison between studies the observed bias in the current study of hip OA patients appears larger than observations in healthy adults [31,33,34,41,42]. Meanwhile, recent validation studies in different elderly patient groups have indicated overestimation during various activities [27,28]. In elderly diabetic patients reported overestimations between 78% and 81% was reported during horizontal walking, which is comparable with the current observations in hip OA patients [28]. In correspondence with observations in healthy adults [34] and the current observations (during stair climb), Machač et al. (2013) also reported underestimation during walking with inclination [28]. The present pronounced overestimation observed in household and garden activities involving upper body movements is likely to rely on the position of the monitor (accelerometer) on the upper arm.

Due to differences in criterion methods there are limitations concerning comparability of studies since the doubly labeled water method used for validation of daily energy expenditure [11,12,30] does not allow for the activity specific validation attended in the current study design. Generally, studies in healthy adults have been performed at higher intensities compared to the current relatively low intensity protocol which may affect the observed validity. Additionally, the protocols differ largely between studies ranging from highly controlled activities (e.g. treadmill walking and RT-exercises) to various degrees of free living or simulated free living protocols. Physiological changes related to ageing has been suggested as a source to discrepancy between studies in elderly and younger adults [24], however the inaccessible inner algorithms deny further analysis of the individual contributions of the various physiological outputs of the SWA unit.

### Clinical implications

The present findings raise concerns regarding the validity in patients with osteoarthritis of the hip, which is important for the application and interpretation of activity monitor estimates within the present patient group, as well as in comparison between patients and healthy subjects. Despite reported high correlation between the SWA and indirect calorimetry the large bias (overestimation) observed during a variety of common activities of daily living represents a major concern for the validity in patients with hip OA. Furthermore, since both bias and variance showed dependency of the type of activity performed, alteration in activities of daily living, as might be expected following surgery or non-surgical interventions may further compromise the validity in clinical use.

### Study limitations

The relative low number of participants limits further subgroup analysis. The number of activities in the protocol is restricted which limits generalization of results regarding actual free living. As the overall intensity in the protocol was rather low and the protocol dictated a number of resting periods in between the activities, the bias of a possible carry-over effect (the physiological delay in obtaining steady state after a change in activity) was assumable low, supported by the observed stable bias during the long resting periods (#1, #7 and #14) placed at the beginning, middle and end of the protocol (Figure 1). The intensity of the activities in the protocol makes the results applicable only to a sedentary lifestyle. However, since none of the participants complied to the highest intensity activity (jogging/running) we believe, the protocol reflected typical activities of daily living for the present patient group and a higher intensity protocol was redundant. The use of a walking aid may affect the outcome of activity monitors during walking activities and the exclusion of patients dependent on walking aid limits the generalization of the result regarding hip OA patients with severe functional impairments. Finally, it is a limitation that the outputs from the various sensors of the SWA are inaccessible for analysis regarding their individual contribution to the outcome and their contribution to the error of measurements since the monitor essentially is a “black box”.

## Conclusion

In patients with hip osteoarthritis the SWA activity monitor showed substantial bias (overestimation) during common activities of daily living, especially when involving the upper body. Despite a high correlation between the activity monitor and indirect calorimetry, the size and direction of bias and variance between methods varied between activities indicating limited validity of the estimations of physical activity in patients with hip osteoarthritis.

In perspectives, for future prospective studies in patients with hip OA (i.e. in cohort or interventional studies), further validation studies of activity monitors and accelometers are needed as this study emphasizes the importance of both patient and apparatus specific validation studies prior to a clinical application.

## Competing interests

The authors declare that they have no competing interests.

## Authors’ contributions

AH: Conception and design, collection of data, analysis and interpretation of the data, drafting of the article, obtaining of funding. MR-L: Conception and design, analysis and interpretation of the data, technical and logistic support. AKJ: Analysis and interpretation of the data, statistical expertise, drafting the article. RH: Analysis and interpretation of the data, statistical expertise. LBA: Conception and design, analysis and interpretation of the data, critical revision of the article for important intellectual content. SO: Conception and design, analysis and interpretation of the data, critical revision of the article for important intellectual content. AH-L: Conception and design, analysis and interpretation of the data, drafting the article, Critical revision of the article for important intellectual content. All authors read and approved the final manuscript.

## Authors’ information

AH: MD, PhD student, Orthopedic Research Unit, Department of Orthopaedic Surgery and Traumatology, Odense University Hospital, Institute of Clinical Research, University of Southern Denmark.

ML: cand. scient, PhD student, Institute of Sport Sciences and Clinical Biomechanics, University of Southern Denmark.

AKJ: cand. polyt, Phd student, Department of biostatistics, Institute of Public Health, University of Southern Denmark.

RH: Associate professor, Department of biostatistics, Institute of Public Health, University of Southern Denmark.

LBA: Professor, Dr.med, Chief of research, Institute of Sport Sciences and Clinical Biomechanics, RICH, Exercise Epidemiology, University of Southern Denmark.

SO: Professor, Dr.med, PhD, University of Southern Denmark, Chief of research, Orthopedic Research Unit, Department of Orthopaedic Surgery and Traumatology, Odense University Hospital, Institute of Clinical Research, Odense University Hospital.

AHL: Associate Professor, PhD, Orthopedic Research Unit, Department of Orthopaedics Surgery and Traumatology, Odense University Hospital.

## Pre-publication history

The pre-publication history for this paper can be accessed here:

http://www.biomedcentral.com/1471-2474/15/43/prepub
